# Patients With Suspected Severe Adverse Reactions to COVID-19 Vaccination Admitted to Intensive Care Unit: A Case Report

**DOI:** 10.3389/fmed.2022.823837

**Published:** 2022-03-18

**Authors:** Denise Battaglini, Lorenzo Ball, Chiara Robba, Simona Maiani, Iole Brunetti, Luana Benedetti, Lucio Castellan, Gianluigi Zona, Giampaola Pesce, Patricia R. M. Rocco, Paolo Pelosi

**Affiliations:** ^1^Anesthesia and Intensive Care, San Martino Policlinico Hospital, IRCCS for Oncology and Neuroscience, Genoa, Italy; ^2^Department of Medicine, University of Barcelona, Barcelona, Spain; ^3^Department of Surgical Science and Integrated Diagnostics (DISC), University of Genoa, Genoa, Italy; ^4^Department of Neurosciences, Rehabilitation, Ophthalmology, Genetics and Maternal and Children (DINOGMI), University of Genoa, Genoa, Italy; ^5^UO Clinica Neurologica, San Martino Policlinico Hospital, IRCCS for Oncology and Neuroscience, Genoa, Italy; ^6^Department of Radiology and Neuroradiology, San Martino Policlinico Hospital, IRCCS for Oncology and Neuroscience, Genoa, Italy; ^7^Department of Neurosurgery, San Martino Policlinico Hospital, IRCCS for Oncology and Neuroscience, Genoa, Italy; ^8^UOSD Laboratorio Diagnostico di Autoimmunologia, IRCCS Ospedale Policlinico San Martino, Genoa, Italy; ^9^Dipartimento di Medicina Interna e Specialità Mediche (DiMI), Università di Genova, Genoa, Italy; ^10^Laboratory of Pulmonary Investigation, Carlos Chagas Filho Biophysics Institute, Federal University of Rio de Janeiro, Rio de Janeiro, Brazil

**Keywords:** COVID-19, vaccines, coronavirus disease 2019, vaccine-induced immune thrombotic thrombocytopenia, thrombosis with thrombocytopenia syndrome

## Abstract

**Background:**

Several cases of adverse reactions following vaccination for coronavirus disease 2019 (COVID-19) with adenoviral vector vaccines or mRNA-based vaccines have been reported to date. The underlying syndrome has been named “vaccine-induced immune thrombotic thrombocytopenia” (VITT) or “thrombosis with thrombocytopenia syndrome (TTS)” with different clinical manifestations.

**Methods:**

We report the clinical course of five patients who had severe adverse reactions to COVID-19 vaccines, either with VITT/TTS, abdominal or pulmonary thrombosis after adenoviral vaccines, or Stevens' Johnson syndrome because of mRNA vaccination, all of whom required admission to the intensive care unit (ICU).

**Conclusions:**

All patients with severe or life-threatening suspected reaction to different types of COVID-19 vaccination required ICU admission. A prompt evaluation of early symptoms and individualized clinical management is needed to improve outcomes.

## Introduction

Since December 2019, severe acute respiratory syndrome coronavirus-2 (SARS-CoV-2) has spread worldwide causing millions of deaths. Advances in prevention have been made by starting a global vaccination campaign. COVID-19 vaccines have been recognized safe and effective preventive agents to fight against the pandemic. The Center for Disease Control and Prevention (CDC) recommends everyone ages 5 y/o and older get vaccinated. However, given the latest evidence about severe adverse reactions to non-mRNA vaccinations, the CDC has updated its recommendation with a preference for mRNA vaccines (1). Indeed, the recombinant adenoviral vector vaccine ChAdOx1-S/nCoV-19 and the recombinant replication-incompetent adenovirus type 26 (Ad26) vectored vaccine ad26.cov2.s have been recognized as possible causative factors of vaccine-induced immune thrombotic thrombocytopenia (VITT) (2–4) and other systemic reactions with potential for life-threatening adverse complications. This syndrome has been also named “vaccine-induced prothrombotic immune thrombocytopenia (VIPIT)”, and ultimately as “thrombosis with thrombocytopenia syndrome (TTS)” (5–7). Similar to vectored vaccines, adverse reactions, although less severe, have also been reported with mRNA-based coronavirus disease-2019 (COVID-19) vaccines (BNT162b2 and mRNA-1273) (8). Data from the drug agencies confirm that the incidence of TTS/VITT is relatively low in the general population, but the incidence is increased in women younger than 50 years (9). Indeed, the incidence of TTS/VITT is relatively low in the general population, of around 15 cases reported following 7.98 million doses administered of ad26.cov2.s, while the incidence in young individuals is increased to 7 per million in the group of women younger than 50 years. Similarly, 13 cases of sinus or cerebral vein thrombosis were reported over 1.6 millions of administered doses of ChAdOx1-S/nCoV-19 (9). TTS/VITT occurs between 4 and 16 days after vaccination (9). TTS is a relatively new disease, but diagnostic criteria are becoming clearer over time (10), although therapeutic possibilities remain limited and need to be further investigated (11). Current evidence do not report severe systemic and life-threatening adverse reactions with mRNA-based vaccines to date, except for anaphylaxis (8).

Hence, a complete understanding of the impact, pathophysiology, diagnosis, and therapy of severe adverse reactions is needed, and scientists must share their experiences through examples, hypotheses, discussion, and informal education to enrich knowledge on this under investigated and evolving phenomenon. We report the clinical course of five patients who manifested severe life-threatening adverse reactions to COVID-19 vaccines requiring admission to the intensive care unit (ICU).

## Methods

The investigation included four cases of suspected TTS after vaccination with ChAdOx1-S/nCoV-19 and one suspected case of Stevens-Johnson syndrome (SJS) after mRNA-1273 vaccination, admitted to the ICU at San Martino Policlinico Hospital (SMPH), IRCCS for Oncology and Neuroscience, Genoa, Italy, between March and June 2021. The study was approved by the Ethics Committee of Liguria Region (497/2021). Informed consent was obtained from the patients' next of kin or, for unconscious patients, from a doctor not involved in the study, following the local regulations. Data on previous comorbidities, age, sex, laboratory and clinical findings, and radiographic images were collected as anonymized records. Heparin PF4-complexes were assessed with the highly sensitive and specific ELISA for detecting H-PF4 antibodies of the IgG class (Asserachrom HPIA – IgG, Stago, Asnières-sur-Seine, France).

## Results

### Case 1

**Figure 1:**
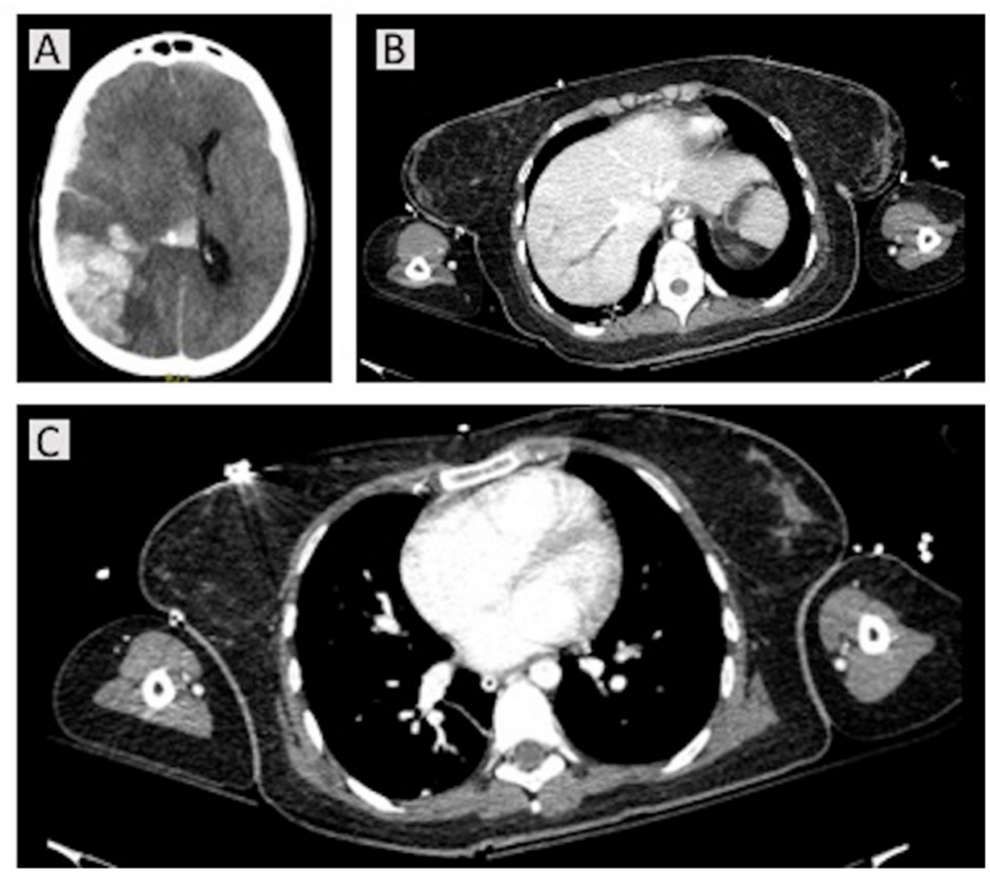
Computed tomography (CT) scans for case 1. A non-contrast brain CT and angio-CT were immediately assessed on hospital admission, showing right temporo-parieto-occipital intraparenchymal hemorrhage with intraventricular hemorrhage, fronto-parietal subdural hematoma, with homolateral fronto-temporal subarachnoid hemorrhage. She presented with intracranial hypertension and a 15-mm midline shift, with uncal, transtentorial, and cerebellar herniations into foramen magnum **(A)**. The homolateral transverse and sigmoid sinuses were completely obliterated, as well as the jugular gulf; the sagittal superior sinus was partially obliterated. The intracranial arteries were obstructed. At thoracic CT perfusion and abdominal scans, pulmonary embolism in the arterial ramifications for the left inferior lobe and increased hepatic volume with thrombosis of the suprahepatic veins were present **(B,C)**.

A young woman aged 32 years was found unconscious (Glasgow Coma Score 3/15 with anisocoria) on 3 April 2021. The patient was admitted to the emergency department (ED) of SMPH. Non-contrast brain computed tomography (CT) imaging and angio-CT were immediately assessed (Figure 1). Laboratory findings on admission are reported in Table 1. The lung CT scan showed ground-glass opacities in the right inferior lobe, and fibrotic thickenings in the left inferior lobe. The lung CT perfusion scan showed pulmonary embolism in the arterial ramifications for the left inferior lobe. The abdominal CT scan showed increased hepatic volume with thrombosis of the suprahepatic veins. The cerebral CT scan showed intraparenchymal hemorrhage with vasogenic edema and midline shift, plus clear signs of cerebral venous sinus thrombosis as depicted in Figure 1. The patient was then sedated, intubated, mechanically ventilated and transferred to the ICU. During the first day in the ICU, the patient became hemodynamically unstable, requiring both anti-hypertensive and aminic support. Mydriasis with unresponsiveness to light appeared and polyuria started. The patient was then transferred again to the CT room for further brain imaging, which showed increased intracranial volume (manifest with refractory intracranial hypertension), hypodensity in the cerebellum, brainstem, cerebral hemispheres, with diffuse parenchymal sufferance. The arterial flow was interrupted at the foramen magnum, and the internal cerebral veins showed no flow, consistent with brain death. Past medical history was negative. Medications included estroprogestinic therapy, ChAdOx1-S/nCoV-19 vaccination 13 days before the thrombotic event.

**Table 1:** Laboratory findings at hospital admission.

**Laboratory findings**	**Case 1**	**Case 2**	**Case 3**	**Case 4**	**Case 5**
Prothrombin, %	65	57	87.6	81	99
Activated partial thromboplastin time, s	54	29.6	36.9	32.8	36.5
Fibrinogen, g/L	1.14	1.88	0.69	1.89	3.77
Antithrombin-III, %	109	84	102	92	69
D-dimer, μg/L	91,673	11,326	37,021	10,393	29,695
Leucocytes, × 10^9^/L	16.09	7.57	8.23	9.20	13.26
Platelets, × 10^9^/L	35	54	40	12	227
Creatinine, mg/dL	0.6	0.7	0.7	0.8	1.5
Total bilirubin, mg/dL	0.96	0.82	0.4	1.03	0.46
Creatine phosphokinase, IU/L	385	42	62	103	118
Pancreatic amylases, IU/L	20	85	9	45	24
Alanine aminotransferase, U/L	27	27	74	20	107
Troponin I, μg/L	0.043	0.289	<0.015	0.260	<0.015
N-terminal fragment of brain natriuretic peptide, ng/L	1,404	108	687	548	NT
C-reactive protein, mg/L	57.5	43.9	19.5	26.9	6.6
Glucose, mg/dL	158	164	132	116	144
Antibodies to platelets factor-4 IgG	Positive	Positive	Positive	NT	NT
ADAMTS-13, %	NT	65	82	0	NT

*NT, not tested; Ig, immunoglobulin*.

### Case 2

A young woman aged 18 years received ChAdOx1-S/nCoV-19 vaccination and 4 days later, she took 2 mg tablets of estradiol valerate and 10 mg of dydrogesterone for an ovarian cyst. Nine days after vaccination, the patient attended the ED of a hospital in Liguria Region for left temporal headache and photosensitivity. During her hospital stay, she underwent a brain CT scan without contrast, which was negative for hemorrhagic lesions, a chest radiograph, and blood chemistry examinations [at the first and second controls, platelet count was 97 × 10^9^/L and 90 × 10^9^/L (normal range, 130–400 × 10^9^/L), glycemia was 119 mg/dL (normal range, 60–110 mg/dL), lymphocyte count was 1.07 × 10^9^/L (normal range, 1.10–4.0 × 10^9^/L)]; the results of other examinations were within normal ranges. The day after, the patient was discharged with a 5-day prognosis and indication of repeating blood cell counts 15 days after hospital discharge.

Two days later, the patient presented with motor hyposthenia on the left side and was readmitted to the same hospital, where a molecular polymerase chain reaction test for SARS-CoV-2 was repeated (negative) and a brain CT scan showed new hemorrhagic hotspots at frontal and left front-basal levels with moderate perilesional edema and hematic spread near grooves at the top. This was associated with initial anterior midline shift and hyper-density in the falx at the top. The patient was transported to the ED of SMPH, sedated, paralyzed, and mechanically ventilated. Levetiracetam for suspected epileptic status, mannitol 18% for intracranial hypertension, and 5 mg of fondaparinux were started.

**Figure 2:**
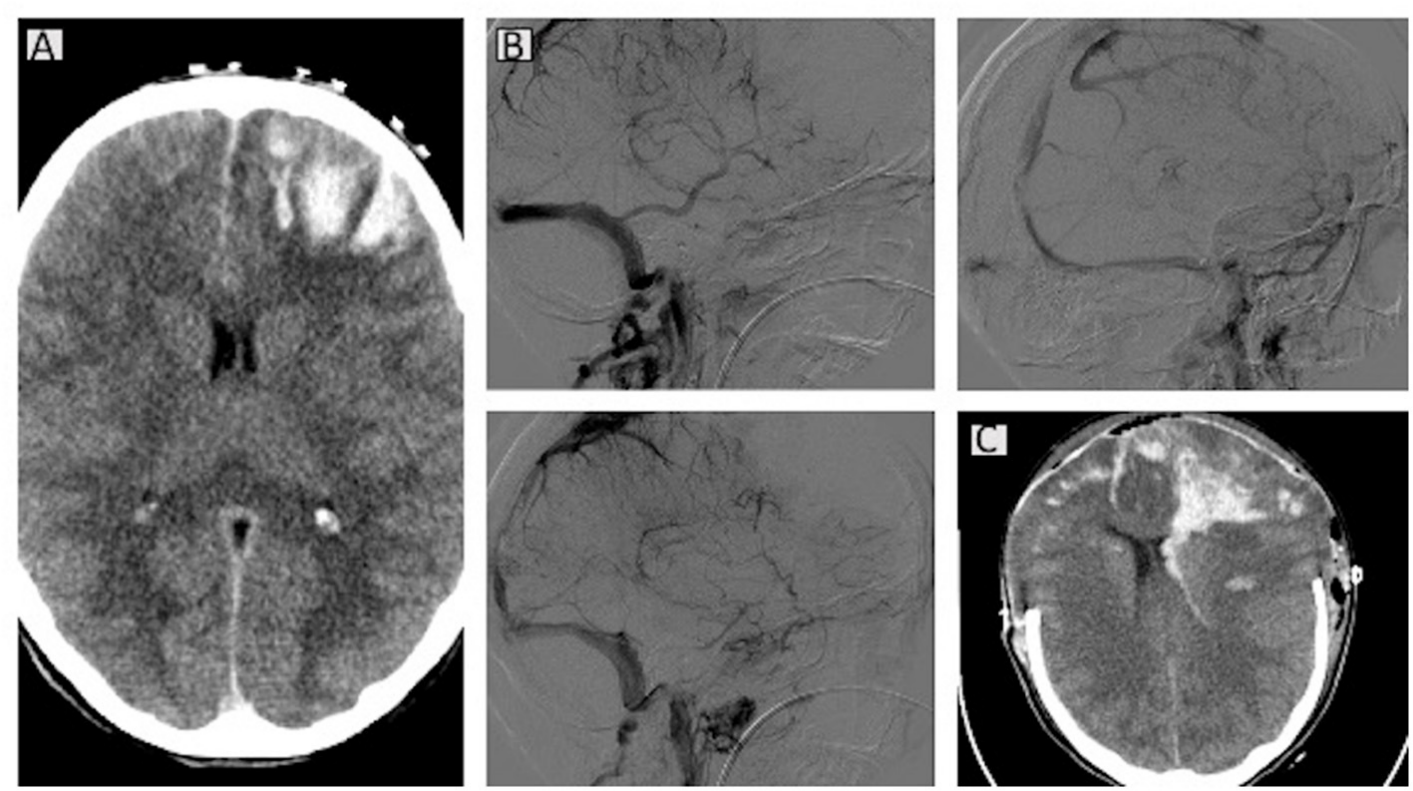
Computed tomography (CT) scans for case 2. A brain CT scan showed bilateral venous infarct with hematic component in the fronto-basal-anterior and parietal areas and associated intraventricular hemorrhage. Initial cerebral herniation of the cerebellar tonsils is present **(A)**. At angio-CT, the arterial circulation is patent, whereas the superior sagittal sinus appears obliterated, the sinus recto is thinner, and the cavernous sinus is partially matted **(B)**. Bilateral decompressive craniectomy and midline shift are evident in the latest CT scan **(C)**.

A new brain CT examination showed marked hyper-density of the superior sagittal sinus and cortical veins, especially at parietal-frontal and vertex levels with suspected of thrombosis (Figure 2). The known hypodensity was increased with hematic filling at cortical and subcortical levels, and around the left frontal anterior white substance and subarachnoid spaces. A hypodense area with hemorrhage at left frontal-basal and right frontal-superior levels was also observed, associated with subarachnoid spread. In addition, a hypodense area of parenchymal distress in the left parietal subcortical area was observed. No midline shift was present, but at angio-CT, the sagittal superior sinus and cortical veins were obliterated with occurrence of hemorrhagic strokes and subarachnoid hemorrhages. After blood sampling for PF4/heparin antibodies, complete blood cell counts, and other routine laboratory examinations, hematologic and neurologic counseling, immunoglobulins and dexamethasone were started. Antibiotic prophylaxis was also administered. Laboratory findings on admission are reported in Table 1.

The patient underwent another CT scan after a few hours, which identified the presence of an increased hematic component with a new hematic spot in the white subcortical substance at frontal and vertex levels with small petechiae in the cortical and subcortical front-anterior-lateral vertex and sub-vertex areas. The dimensions of the subarachnoid spaces were reduced. The results of the blood examinations were as follows: activated partial thromboplastin time (aPTT), 22.1 s [range, 28–40 s]; D-dimer, 14,514 μg/L [range, 0–500 μg/L]; platelet count, 76 × 10^9^/L [range, 130–400 × 10^9^/L]; glycemia, 187 mg/dL, and C-reactive protein, 33.1 mg/L [range, 0–35 mg/L]. She therefore underwent urgent cerebral angiography with therapeutic venous mechanical thrombectomy. After that, in the light of refractory intracranial hypertension, a surgical decompressive craniectomy was done, and hemodynamic support with norepinephrine and transfusions was given. The next day, the patient was admitted to the ICU for multiparametric monitoring and support of vital functions. The patient presented refractory intracranial hypertension (>30–35 mmHg despite maximized therapy), although international guidelines were followed (12, 13).

The patient continued mechanical ventilation, aminic support, and comprehensive critical care until further cerebral deterioration was detected with unreactive pupils, indifferent Babinski reflex, although symmetric and adequate cerebral blood flow was evident at transcranial Doppler (TCD) bilaterally in the mean cerebral arteries. A contrast brain CT and angio-CT showed extension of the venous infarcts with associated hematic component at front-basal/anterior-frontal/ and parietal levels bilaterally. In particular, the hematic component was increased in the frontal-left side with extension to the homolateral lateral ventricle, and a little spot also at the fourth ventricle. Sphenoidal corns were increased in volume, and the subarachnoid spaces were undetectable both at supratentorial and sub-tentorial levels because of edema. Cerebellar tonsils were at a lower extent than normal. Common carotid arteries, internal carotid arteries, and vertebral arteries were patent on angio-CT images. The superior sagittal sinus was still occluded except at the posterior level, and the rectus sinus was thinner. The traverse and sigmoidal sinuses, the jugular veins, Galeno's vein, and the internal cerebral veins were patent, whereas the cavernous sinus was partially obliterated.

The next day, at TCD blood flow velocities worsened bilaterally, despite maximized supportive therapies and continuous non-invasive and invasive neuromonitoring (including continuous electroencephalography, automated pupillometry, TCD, and invasive intracranial pressure monitoring), recovery from refractory intracranial hypertension was not achieved, and brain death occurred within a few days. Medical history included familiar autoimmune thrombocytopenia and ovarian cyst. Medications included estradiol valerate and progestogen therapies, ChAdOx1-S/nCoV-19 vaccination around 10 days before the thrombotic events.

### Case 3

A 34-year-old woman was admitted to the ED for headache and thoracic pain. On admission, she underwent brain, thoracic, and abdominal CT scans, which showed evidence of pulmonary embolism of the lobar branches of the left and right inferior lobes, and extensive suprahepatic and intrahepatic thrombosis of the portal ramifications for the left fourth, second, third hepatic segments. The patient was sedated, intubated, mechanically ventilated to safely receive a transjugular intrahepatic portosystemic shunt (TIPS) with local injection of 100,000 IU of urokinase in the portal ramification for the left fourth segment, and 2,00,000 IU in the left portal branch, followed by intravenous (IV) continuous infusion of 70,000 IU/h every 12 h. After collecting blood samples for analysis, fondaparinux 7.5 mg subcutaneously, dexamethasone IV 20 mg, and intravenous immunoglobulin (IVIG) 1 g/kg/day for 2 days were started, and the patient was transferred to the ICU. One day later, the patient was gradually awakened and finally extubated. Within a few days, the patient was discharged from the ICU and then from the hospital. Medical history included microcytic anemia. She was not on any medications other than ChAdOx1-S/nCoV-19 vaccination around 10 days before the thrombotic events.

### Case 4

A 43-year-old woman was admitted to the ED with neurologic symptoms (right hemiparesis and hemianopsia with global aphasia) and skin manifestations similar to petechiae. A CT scan showed left anterior subinsular hypodensity; the venous sinuses were patent on angio-CT images, and bilateral coiling of the internal carotids was detected. After acquisition of radiographic images, the patient was transferred to the neurologic medical unit and 250 mg of acetylsalicylate lysine was administered. The patient was then admitted to the ICU to treat the diagnosed thrombotic thrombocytopenic purpura, after appropriate blood sampling and a peripheral blood smear where schistocytes were found without myeloid alterations. The patient was given caplacizumab 10 mg intravenously followed by plasmapheresis, and then continued at the dosage of 10 mg subcutaneously after each session. In addition, methylprednisolone 1 mg/kg every day and rituximab 375 mg/m^2^ every day for 2 days were administered intravenously. The patient was discharged from the hospital after a few weeks. Medical history included anemia with thalassemic trait (previous transfusions), estroprogestinic therapy, smoking habit, previous pregnancy. She was not on any medications other than ChAdOx1-S/nCoV-19 vaccination around 7 days before the event.

### Case 5

A 67-year-old man was admitted to the ED after mRNA-1273 vaccination. He had facial erythema consisting of individual blemishes that looked like a target on his face, thorax, arms, excluding his palms (darker in the middle and lighter around) with some blistered and ulcerated lesions. SJS was suspected. The patient was transferred immediately to our ICU for possible systemic deterioration and quick initiation of treatment. During his ICU stay, the patient did not need airway management and multiorgan functions were normal. Oculist and dermatologic examinations confirmed SJS. Intravenous hydration and medication of the lesions were assessed daily until continuous improvement.

## Discussion

We have presented four cases of suspected TTS/VITT after ChAdOx1-S/nCoV-19 vaccination of patients who needed admission to the ICU for severe or life-threatening conditions. The findings confirmed that: (1) the patients received ChAdOx1-S/nCoV-19 vaccine from 4 to 30 days before the event; (2) all the patients presented radiologic evidence of venous or arterial thrombosis; and (3) all were positive for PF4-heparin antibodies; (4) all had a reduced platelet count. Moreover, we reported one case of SJS suspected to be a reaction to mRNA-1283 vaccination; that patient required critical care and ICU admission.

Cases of adverse reactions to COVID-19 vaccine who require ICU admission are increasingly being reporting in the literature, although most presentations do not require critical care (14–16) (Table 2). We discuss the status of international regulations, pathophysiology, diagnostic criteria and treatment possibilities for managing severe cases of adverse reactions to COVID-19 vaccination.

**Table 2:** Reported systemic manifestations of COVID-19 vaccines.

	**ChAdOx1-S/nCoV-19**	**ad26.cov2.s**	**BNT162b2**	**mRNA-1273**
Systemic reactions (mild)	Tenderness, injection site pain, headache, fatigue, myalgia, malaise, pyrexia, fever, chills, arthralgia, nausea	Injection site pain, headache, fatigue, myalgia, nausea, fever	Fever, fatigue, headache, chills, muscle pain, joint pain, vomiting, diarrhea	Injection site pain, fatigue, headache, myalgias, arthralgias, chills, nausea, vomiting, fever
Systemic reactions (moderate)	Lymphadenopathy, Bell's palsy	Lymphadenopathy	Lymphadenopathy, Bell's palsy	Lymphadenopathy, Bell's palsy
**Systemic reactions (severe)**				
Gastrointestinal	Splanchnic thrombosis, abdominal pain, intestinal bleeding	Splanchnic thrombosis, abdominal pain	Appendicitis	Not clearly reported
Reno-vascular	Angiitis hypersensitiva, Schonlein-Henochs purpura, Buerger's syndrome, Goodpasture syndrome, microangiopathy thrombotic, necrotizing vasculitis, thrombotic thrombocytopenic purpura, hematuria	Rhabdomyolysis, acute kidney injury	Not clearly reported	Not clearly reported
Cardiovascular	Acute myocardial infarction, ischemic heart disease without infarction, deep thrombophlebitis	Myocarditis, pericarditis, atrial flutter, deep thrombophlebitis	Acute myocardial infarction	Myocarditis, pericarditis
Respiratory	Pulmonary thromboembolism, bleeding from the respiratory tract	Pulmonary thromboembolism	Not clearly reported	Not clearly reported
Neurologic	TTS, cerebrovascular accident and sinus thrombosis, GBS, transverse myelitis, intracerebral hemorrhage	TTS, cerebrovascular accident and sinus thrombosis, GBS, transverse myelitis, intracerebral hemorrhage, seizures	Cerebrovascular accident	Lipothymias, vagal reactions
Coagulative/ hematologic	Thrombocytopenia, bleeding	Thrombosis with thrombocytopenia, immune thrombocytopenic purpura	Not clearly reported	Not clearly reported
Other	Capillary leak syndrome, hypersensitivity, anaphylaxis	Hypersensitivity, anaphylaxis	Hypersensitivity, anaphylaxis	Hypersensitivity, anaphylaxis, erythema multiforme

*Systemic mild, moderate, and severe reactions reported after administration of COVID-19 vaccines*.

*GBS, Guillan Barrè syndrome; TTS, thrombosis with thrombocytopenia syndrome*.

### International Regulations

The US Food and Drug Administration and the Centers for Disease Control and Prevention in the United States paused approval for the administration of the ad26.cov2.s vaccine to investigate the occurrence of various cases of severe thrombocytopenia with thrombosis that occurred after vaccination. Similarly, the European Medicines Agency (17), gathering data on 20 million people vaccinated in the United Kingdom, temporarily stopped administration of ChAdOx1-S/nCoV-19 in European countries (9), concluding that the number of events after vaccination was lower than the estimated rate of similar events in the general population (17). Finally, the COVID-19 subcommittee of the World Health Organization, confirmed a possible causal correlation between vaccinations and the occurrence of thrombosis (18). On 19 July 2021, the World Health Organization published the first report on managing cases of suspected TTS/VITT/VIPT (19). However, some countries are still administering these vaccines, but now with age-specific recommendations.

### Pathogenesis of Adverse Reactions to COVID-19 Vaccines

The Society of Thrombosis and Hemostasis Research has summarized the possible photogenetic mechanism underlying TTS disease. After vaccination, inflammatory and immune mechanisms may occur likely to induce the formation of antibodies against platelet antigens, which causes massive platelet activation by binding to the Fc receptor (20). Several hypotheses have been highlighted in patients who have never encountered SARS-CoV-2 and in those who have already been infected by the virus. Patients who never tested positive for SARS-CoV-2 infection underwent TTS/VITT because of the following mechanisms (21): IgG anti-PF4/heparin antibodies present; no proximate exposure to heparin, “severe” thrombocytopenia; temporal association (events observed 14 days after vaccination); FcɤRIIa-dependent platelet activation (resembles “spontaneous” or “autoimmune” HIT). Those who tested positive for SARS-CoV-2 may develop high levels of IgG anti-PF4 antibodies; IgG anti-PF4/heparin antibodies were not detected; COVID-19 patients were reported positive with a platelet serotonin-release assay even in absence of heparin; PF4 can bind to polyanionic structures (DNA/RNA), which are released from viruses; some IgG antibodies directed against the S protein may be able to interact with FcɤRIIa; possible platelet apoptosis via cross-linking with FcɤRIIa; formation of immune complexes containing IgG might trigger platelet activation; and the interaction between SARS-CoV-2 and platelets can lead to P-selectin secretion and neutrophil activation (NETosis). Moreover, some patients may manifest reactions such as reactivation of a previous immune response producing antibodies directed against the receptor binding domain of the S protein, thus activating platelets without addition of heparin *in vitro*; or even vaccinated patients may develop an immune response before vaccination (due to other activators such as pregnancy); or the vaccine may reactivate previous immunization against PF4 (21). In addition, other mechanisms include the activation of platelets by a viral vector (e.g., Adenoviruses) possibly leading to platelet activation; release of apoptosis-like markers; activated platelets release adenosine-diphosphate, polyphosphate, or serotonin; or polyphosphate/PF4 complexes can mediate heparin-platelet activation in HIT. Finally, the latest possible mechanisms include the activation of other cell types (monocytes, macrophages, and endothelial cells express FcɤRIIa, which can be activated by immune complexes after vaccination, triggering a proinflammatory and procoagulant state). Moreover, the generation of thrombin is downregulated in extracerebral vessels because of very low levels of thrombomodulin (21).

SJS is an inflammatory mucocutaneous reaction to certain drugs, infections, and other causes, that can manifest as flu-like symptoms and erythema multiforme, characterized by targetoid cutaneous lesions comprising <10% of the body surface area, associated with toxic epidermal necrolysis with mucocutaneous involvement affecting more than 30% of the skin surface. SJS has been reported as a rare manifestation after vaccination (22, 23). To date, no clear evidence exists about the possible manifestation of SJS after COVID-19 vaccination because only one case has been reported in the literature (24, 25). The exact mechanism of SJS is unknown, but several hypotheses have been suggested. Findings suggest that the cytotoxic T cells and natural killer lymphocytes may release granulysin, which causes keratinocyte death; also, IL-15 has been found in patients with SJS with increasing granulysin production. Another theory involves the interactions between a cell-surface receptor (Fas) and its ligand released from mononuclear cells, thus leading to apoptosis and necrosis. In addition, a genetic predisposition has been hypothesized (26). SJS has also been identified in patients with SARS-CoV-2 infection or treated with hydroxychloroquine, raising interesting questions about a possible pathogenetic mechanism (27, 28).

### Diagnostic Criteria

The diagnostic criteria for TTS/VITT are becoming clearer over time (19). The definition of TTS is based on the combined presence of a thrombosis and new onset thrombocytopenia, stratified on three levels of certainty based on the anatomic location of the thrombosis and the severity of thrombocytopenia (19).

**Figure 3:**
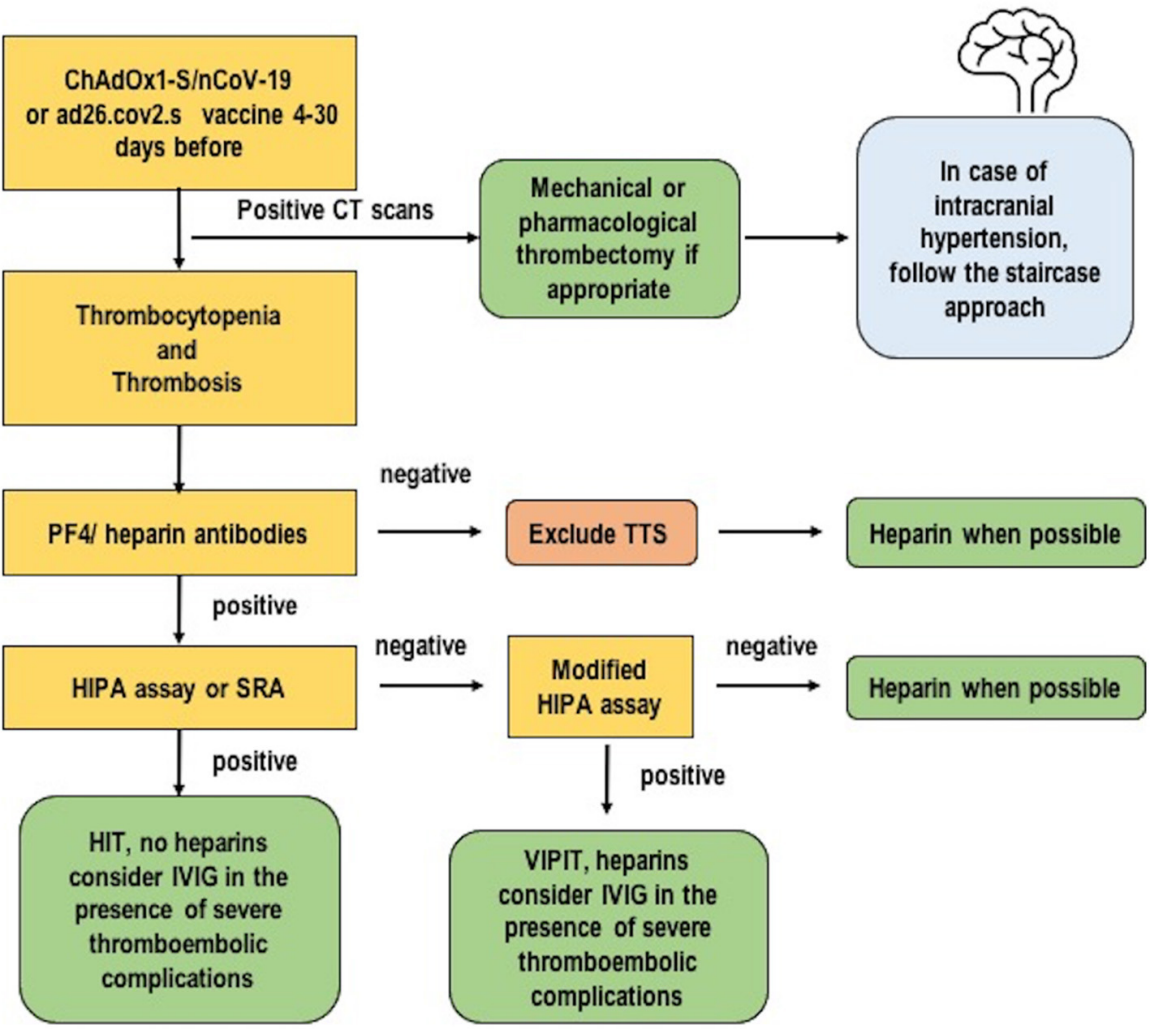
A possible diagnostic and therapeutic algorithm for thrombosis with thrombocytopenia syndrome. Modified from Oldenburg et al. (9).

Our cases were tested for PF4 antibodies with confirmed positivity for PF4 antibodies as expected (29). With regard to the clinical criteria, TTS/VITT is suspected in cases of severe headache, visual changes, abdominal pain, nausea and vomiting, back pain, shortness of breath, leg pain or swelling, petechiae, easy bruising, or bleeding in individuals who have received a dose of ad26.cov2.s or ChAdOx1-S/nCoV-19 vaccine after 24 to 48 h. In cases of recurrent or persistent symptoms within 30 days of vaccination, with no alternative explanation for the condition (i.e., no heparin administration in the previous 100 days) and a combination of thrombosis and thrombocytopenia, TTS should be considered (19). TTS is diagnosed if four criteria are met contemporaneously: (1) COVID-19 vaccine administered from 4 to 30 days before the event; (2) venous or arterial thrombosis (often cerebral or splanchnic); (3) thrombocytopenia (<150×10^9^/L or <50% from baseline); and (4) positive PF4 HIT on ELISA in the absence of heparin (10, 29, 30). Minor criteria include thrombosis of minor/other arteries with or without specific a clinical syndrome in any location and platelet count >150 × 10^9^/L or decreased more than 50% from baseline with D-dimer >4,000 μg/L (19). Ancillary tests should include a complete blood count with platelet count, imaging for thrombosis, complete coagulation including fibrinogen and D-dimer. The differential diagnosis includes HIT (in the presence of heparin) and HIT-like autoimmune thrombosis (30). Oldenburg et al. (9, 20) produced a diagnostic and therapeutic algorithm to manage a patient with suspected VITT. A modified version of that checklist is proposed in Figure 3.

Diagnostic criteria for SJS are based on clinical grounds and eventual skin biopsy to exclude other diseases such as toxic shock syndrome, erythroderma, paraneoplastic lesions, or staphylococcal skin syndrome (26). Skin biopsy usually reveals vacuolization keratinocytes in the basal layer associated with lymphocytes and dermal-epidermal junction and necrotic spinous layer keratinocytes (26), as demonstrated in our patient.

### Treatment Possibilities

To date, no specific treatment is available. In cases of confirmed TTS that require ICU admission, patients should be treated by adopting a strategy that includes protective mechanical ventilation, hemodynamic stability, adequate fluid balance, prevention of infectious disease, prevention and treatment of intracranial hypertension, seizures and brain dysfunction, maintenance of adequate oxygenation, carbon dioxide levels, diuresis, hepatic and renal function, and nutritional status. All these therapeutic strategies should be weighted and individualized on the patient response to a critically ill condition. When considering the ICU management of TTS/VITT, it should account the potential for multiple-organ involvement (31). Indeed, despite TTS represents a rare and localized complication of COVID-19 vaccination, cerebral derangement can also be followed by systemic and life-threatening manifestations as consequence of the brain- organs crosstalk. Brain-organ crosstalk plays a crucial role in physiological and pathological processes in patients who are critically ill. In the brain, the mechanism underlying interorgan crosstalk includes physiological reflexes such as baroreceptors, chemoreceptors, Bain-bridge reflex, Cushing reflex, Hering-Breuer reflex, and the release of mediators such as hormones and inflammatory markers. When the physiological crosstalk is affected, this may lead to serial organ failure, including cardiovascular collapse, kidney injury, gastrointestinal barrier derangement, and more susceptibility to infections (31–33).

Specific treatment may include: (1) IVIG 1 g/kg every day for 2 days or 0.4 g/kg for 5 days (19). In case of inadequate response, a second dose after 2–3 days should be considered. This treatment option accounts the potential for IVIG to competitively inhibits the interaction of TTS antibodies with the platelet FcγIIa receptor thus reducing platelets activation (34). In any case, if IVIG therapy after the second dose of IVIG is not affective, corticosteroids, plasma exchange, or rituximab can be considered (35); (2) direct thrombin inhibitors (argatroban/ bivalirudin) IV or direct oral anticoagulants or fondaparinux or danaparoid; do not use heparin (19). Following most updated recommendations, when using argatroban as an anticoagulation treatment for people with TTS, switch to fondaparinux or a direct oral anticoagulant as soon as the bleeding risk has reduced. Moreover, in case neurosurgery to treat the thrombosis is not planned, oral anticoagulation can be switched to direct anticoagulants if clinically indicated. In case of very low platelets count (<30 × 10^9^/L), a critical illness dose of argatroban or a therapeutic dose plus platelets transfusion can be considered (35); (3) in the case of a low fibrinogen level, fibrinogen replacement should be considered (at least 1.5 g/L); the same applies for IV transfusion of platelets; (4) no recommendation is available on aspirin and plasma exchange, although promising efficacy has been obtained with eculizumab; (5) surgical treatment (3); (6) no platelet transfusion in severe cases (i.e., need surgery, emergency situation) is recommended (19). The role of plasmapheresis is debated (3, 19), although it can be now considered for patients with very poor prognosis (35). Because TTS is Ig-mediated and the mechanism of action of therapeutic plasma exchange is the removal or neutralization of antibodies, this strategy of replacement of fluid would not elevate IgG to inhibitory levels (36).

SJS has a high potential for mortality. The SCORTEN index has been developed to stratify the severity of illness: the higher the score, the more severe the disease. First, recognition and diagnosis are essential to plan a complete and effective multidisciplinary approach. Then, patients with extensive cutaneous involvement should be treated as a burn patient, with proper management of IV administration of fluids, nutritional support, and to prevent infections. The use of medications, including corticosteroids, is controversial, although IVIG has offered promising results (26, 37, 38). Again, like for TTS, the use of IVIG in SJS could be considered since IVIG interact with the effector function of T cells, B cells, and monocytes blocking the interaction of Fas (CD95) with its ligand (FasL, CD95L) (39).

### Patient Perspective

According to current evidence, COVID-19 vaccines are safe and effective. The reported adverse reactions are commonly identified by self-limited mild reactions (i.e., fever, injection site pain, tiredness) that can be interpreted as a confirmation that the body is reacting to vaccination and is building protection. However, severe adverse events have been reported, thus increasing fear and suspicious among citizens. For this reason, the most impactful global organizations and drug agencies timely provide updates on serious adverse events of interest, and have concluded and declared a preference for mRNA vaccines (1). Despite the identification of rare and limited adverse reactions after administration of COVID-19 vaccines (especially ChAdOx1-S/nCoV-19 and ad26.cov2.s), the global effort to produce and distribute vaccines should continue, always highlighting developmental breakthroughs and safety concerns of COVID-19 vaccination, while limiting wrong and conditioning messages to the public.

## Conclusions

All patients with a severe or life-threatening reaction suspected to be due to different types of COVID-19 vaccines required ICU admission. Prompt evaluation of early symptoms and individualized gender-based clinical management and monitoring in the hospital are needed to improve outcomes.

## Data Availability Statement

The raw data supporting the conclusions of this article will be made available by the authors, without undue reservation.

## Ethics Statement

The studies involving human participants were reviewed and approved by Ethic Committee of Liguria Region, Italy (497/2021). Informed consent was obtained directly from the patient, either before the study or retrospectively in case the patient is unconscious at the time of enrolment. If the patient is unable to provide a consent form upon admission, informed consent was obtained by patient next of kin or a doctor not involved in the study.

## Author Contributions

DB drafted the manuscript. LBa, CR, IB, SM, LBe, LC, GP, GZ, PR, and PP revised and edited the text for intellectual content and approved the submitted version. All authors contributed to the article and approved the submitted version.

## Conflict of Interest

The authors declare that the research was conducted in the absence of any commercial or financial relationships that could be construed as a potential conflict of interest.

## Publisher's Note

All claims expressed in this article are solely those of the authors and do not necessarily represent those of their affiliated organizations, or those of the publisher, the editors and the reviewers. Any product that may be evaluated in this article, or claim that may be made by its manufacturer, is not guaranteed or endorsed by the publisher.
